# A standardized framework for representation of ancestry data in genomics studies, with application to the NHGRI-EBI GWAS Catalog

**DOI:** 10.1186/s13059-018-1396-2

**Published:** 2018-02-15

**Authors:** Joannella Morales, Danielle Welter, Emily H. Bowler, Maria Cerezo, Laura W. Harris, Aoife C. McMahon, Peggy Hall, Heather A. Junkins, Annalisa Milano, Emma Hastings, Cinzia Malangone, Annalisa Buniello, Tony Burdett, Paul Flicek, Helen Parkinson, Fiona Cunningham, Lucia A. Hindorff, Jacqueline A. L. MacArthur

**Affiliations:** 1 European Molecular Biology Laboratory, European Bioinformatics Institute, Wellcome Genome Campus, Hinxton, Cambridge, CB10 1SD UK; 2 Division of Genomic Medicine, National Human Genome Research Institute, National Institutes of Health, Bethesda, MD 20892-9305 USA

**Keywords:** Genomics, Genome-wide association studies, GWAS Catalog, Ancestry, Diversity, Population genetics

## Abstract

The accurate description of ancestry is essential to interpret, access, and integrate human genomics data, and to ensure that these benefit individuals from all ancestral backgrounds. However, there are no established guidelines for the representation of ancestry information. Here we describe a framework for the accurate and standardized description of sample ancestry, and validate it by application to the NHGRI-EBI GWAS Catalog. We confirm known biases and gaps in diversity, and find that African and Hispanic or Latin American ancestry populations contribute a disproportionately high number of associations. It is our hope that widespread adoption of this framework will lead to improved analysis, interpretation, and integration of human genomics data.

**Electronic supplementary material:**

The online version of this article (10.1186/s13059-018-1396-2) contains supplementary material, which is available to authorized users.

## Background

The past 15 years have seen a dramatic growth in the field of genomics, with numerous efforts focused on understanding the etiology of common human disease and translating this to advances in the clinic. Essential to the interpretation of this vast amount of data is the accurate and unambiguous description of the ancestry of samples. Degrees of genetic diversity and patterns of linkage disequilibrium (LD) vary by ancestry, with implications for the generalizability of results and the identification of disease-causing variants. The standardized representation of ancestry data is also indispensable to facilitate data access in bioinformatics resources and to support the integration of information from different sources, ultimately enabling more robust analyses of “big data” sets. The need for genetic studies in more ancestrally diverse populations has been repeatedly articulated [1], most recently by Popejoy and Fullerton [2]. Although inclusion efforts are improving over time, it is challenging to assess the status of such efforts without a standardized way of representing ancestry data.

There are currently no established guidelines for the description of ancestral information. We here provide a framework to represent, in an accurate and standardized manner, the ancestry of samples included in human genomics studies. We utilize our method to describe samples analyzed in over 3200 publications included in the NHGRI-EBI GWAS Catalog [3–5], validating its applicability to large and complex data sets. We also present a new and expanded analysis of Catalog ancestry content using, for the first time, our standardized framework. We thus demonstrate the efficacy of categories to facilitate data analysis, including tracking trends in the area of diversity. Finally, to ensure broader applicability beyond the Catalog to other studies or resources involving human subjects, we offer recommendations to authors and provide an ancestry-specific ontology for application to bioinformatics resources. We also apply our method to the 1000 Genomes [6] and HapMap [7] project populations to enable integration with any samples described utilizing these well-established reference populations and of any variation data generated from these projects.

## Results

### Ancestry framework

Our framework involves representing the ancestry of samples in two forms: (1) a detailed description and (2) an ancestry category from a controlled list (Table 1). Detailed descriptions aim to capture accurate, informative, and comprehensive information regarding the ancestry or genealogy of each distinct sample. Category assignment reduces complexity within data sets and enables the establishment of hierarchical relationships, placing samples in context with other samples, groups, and populations. This is extremely useful, empowering more precise search functionalities and improved access to data in bioinformatics resources. This process also facilitates integration of results from multiple sources, ultimately enabling the community to better interpret findings and perform further analyses.

**Table 1:** Ancestry categories: distinct regional population groupings used in this framework

Ancestry category	Definition	Examples of detailed descriptions for samples included in the category
Aboriginal Australian	Includes individuals who either self-report or have been described by authors as Australian Aboriginal. These are expected to be descendants of early human migration into Australia from Eastern Asia and can be distinguished from other Asian populations by mtDNA and Y chromosome variation [29, 30]	Martu Australian Aboriginal
African American or Afro-Caribbean	Includes individuals who either self-report or have been described by authors as African American or Afro-Caribbean. This category also includes individuals who genetically cluster with reference populations from this region, for example, 1000 Genomes and/or HapMap ACB or ASW populations. We note that there is likely to be significant admixture with European ancestry populations	African American, African Caribbean
African unspecified	Includes individuals that either self-report or have been described as African, but there was not sufficient information to allow classification as African American, Afro-Caribbean or Sub-Saharan African	African, non-Hispanic black
Asian unspecified	Includes individuals that either self-report or have been described as Asian but there was not sufficient information to allow classification as East Asian, Central Asian, South Asian, or South-East Asian	Asian, Asian American
Central Asian	Includes individuals who either self-report or have been described by authors as Central Asian [31]. We note that there does not appear to be a suitable reference population for this population and efforts are required to fill this gap	Silk Road (founder/genetic isolate)
East Asian	Includes individuals who either self-report or have been described by authors as East Asian or one of the sub-populations from this region (e.g., Chinese). This category also includes individuals who genetically cluster with reference populations from this region, for example, 1000 Genomes and/or HapMap CDX, CHB, CHS, and JPT populations	Chinese, Japanese, Korean
European	Includes individuals who either self-report or have been described by authors as European, Caucasian, white, or one of the sub-populations from this region (e.g., Dutch). This category also includes individuals who genetically cluster with reference populations from this region, for example, 1000 Genomes and/or HapMap CEU, FIN, GBR, IBS, and TSI populations	Spanish, Swedish
Greater Middle Eastern (Middle Eastern, North African, or Persian)	Includes individuals who self-report or were described by authors as Middle Eastern, North African, Persian, or one of the sub-populations from this region (e.g., Saudi Arabian) [32]. We note there is heterogeneity in this category with different degrees of admixture as well as levels of genetic isolation. We note that there does not appear to be a suitable reference population for this category and efforts are required to fill this gap	Tunisian, Arab, Iranian
Hispanic or Latin American	Includes individuals who either self-report or are described by authors as Hispanic, Latino, Latin American, or one of the sub-populations from this region. This category includes individuals with known admixture of primarily European, African, and Native American ancestries, though some may have also a degree of Asian (e.g., Peru). We also note that the levels of admixture vary depending on the country, with Caribbean countries carrying higher levels of African admixture when compared to South American countries, for example. This category also includes individuals who genetically cluster with reference populations from this region, for example, 1000 Genomes and/or HapMap CLM, MXL, PEL, and PUR populations [17, 33]	Brazilian, Mexican
Native American	Includes indigenous individuals of North, Central, and South America, descended from the original human migration into the Americas from Siberia [34]. We note that there does not appear to be a suitable reference population for this category and efforts are required to fill this gap	Pima Indian, Plains American Indian
Not reported	Includes individuals for which no ancestry or country of recruitment information is available	
Oceanian	Includes individuals that either self-report or have been described by authors as Oceanian or one of the sub-populations from this region (e.g., Native Hawaiian) [35]. We note that there does not appear to be a suitable reference population for this category and efforts are required to fill this gap	Solomon Islander, Micronesian
Other	Includes individuals where an ancestry descriptor is known but insufficient information is available to allow assignment to one of the other categories	Surinamese, Russian
Other admixed ancestry	Includes individuals who either self-report or have been described by authors as admixed and do not fit the definition of the other admixed categories already defined (“African American or Afro-Caribbean” or “Hispanic or Latin American”)	
South Asian	Includes individuals who either self-report or have been described by authors as South Asian or one of the sub-populations from this region (e.g., Asian Indian). This category also includes individuals who genetically cluster with reference populations from this region, for example, 1000 Genomes and/or HapMap BEB, GIH, ITU, PJL, and STU populations	Bangladeshi, Sri Lankan Sinhalese
South East Asian	Includes individuals who either self-report or have been described by authors as South East Asian or one of the sub-populations from this region (e.g., Vietnamese). This category also includes individuals who genetically cluster with reference populations from this region, for example, 1000 Genomes KHV population. We note that East Asian and South East Asian populations are often conflated. However, recent studies indicate a unique genetic background for South East Asian populations	Thai, Malay
Sub-Saharan African	Includes individuals who either self-report or have been described by authors as Sub-Saharan African or one of the sub-populations from this region (e.g., Yoruban). This category also includes individuals who genetically cluster with reference populations from this region, for example, 1000 Genomes and/or HapMap ESN, LWK, GWD, MSL, MKK, and YRI populations	Yoruban, Gambian

Ancestry categories are assigned to samples with distinct and well-defined patterns of genetic variation, in addition to individuals with inferred relatedness to these samples. A full list of GWAS Catalog sample descriptions assigned to each category can be found in Additional file 3: Table S2

### Validation in the NHGRI-EBI GWAS Catalog

To validate the framework, we applied our method to all publications included in the GWAS Catalog—3200 publications, representing 4600 separate GWA studies, 60000 associations, and 110 million individuals, as of November 2017. The Catalog is widely used, and invaluable for researching existing findings on common diseases and supporting investigations to identify causal variants, understand disease mechanisms, and establish targets for treatment [8–11]. As one of the largest repositories and visual summaries of genomic association data, the Catalog provided an ideal substrate on which to test our method and its applicability to large and complex data sets.

**Fig. 1:**
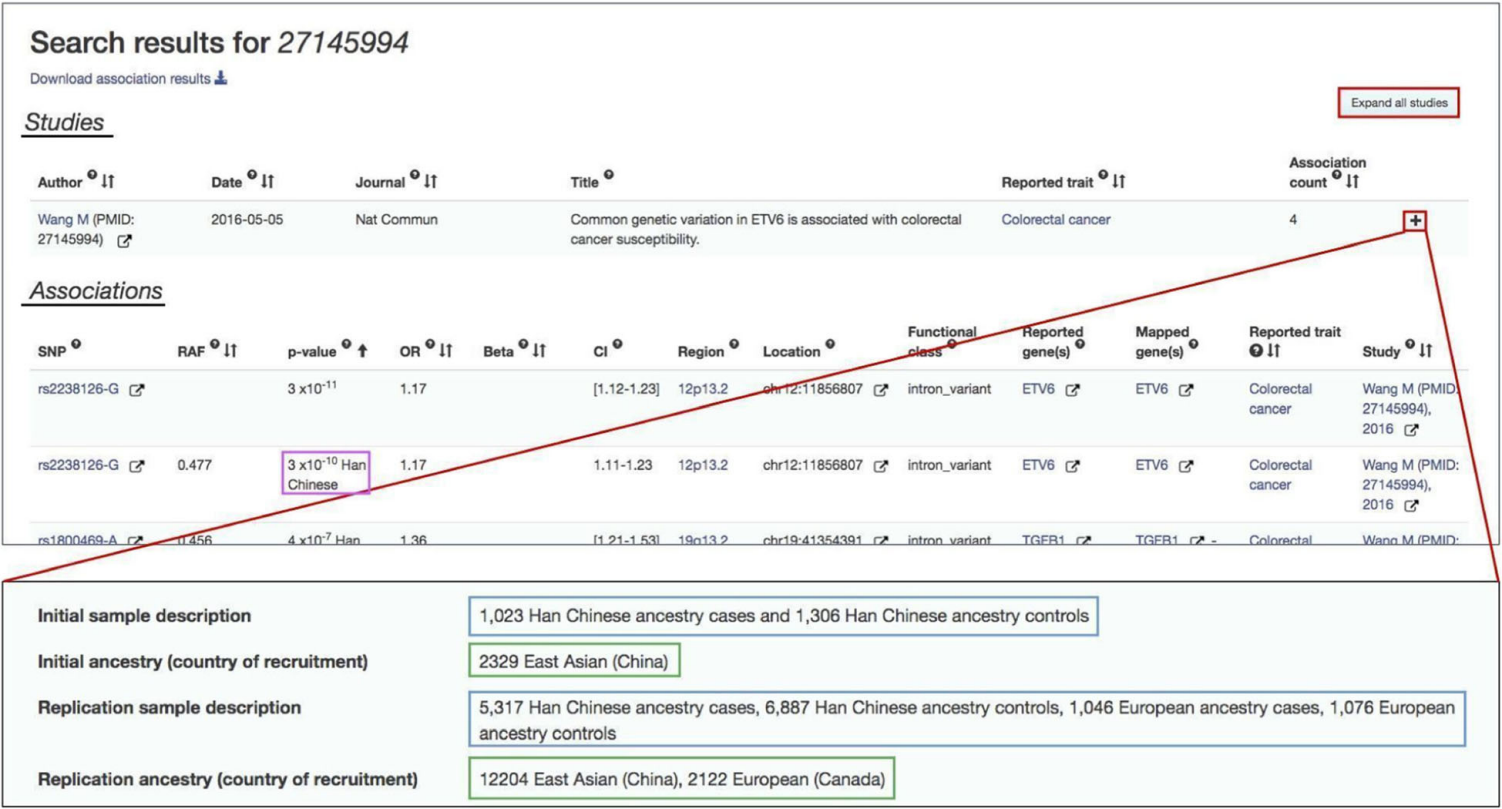
Representation of ancestry data in the GWAS Catalog search interface (https://www.ebi.ac.uk/gwas/). Ancestry-related data are found in the Studies and Associations tables (underlined in *black*) when searching the Catalog. This figure shows the results of a search for PubMed Identifier 27145994. The sample description can be found in the Studies table, either by pressing “Expand all Studies” or the “+” on the study of interest (highlighted in *red*). Sample ancestry is captured in two forms: (1) detailed description (highlighted in *blue*); and (2) ancestry category (highlighted in *green*). The latter follows the format: sample size, category, (country of recruitment). In cases where multiple ancestries are included in a study, the ancestry associated with a particular association is found as an annotation in the *p* value column in the Associations table (highlighted in *pink*)

Each Catalog study entry comprises one or more samples, designated as “Initial” or “Replication” samples, depending on the stage of the GWA study in which they were analyzed (Fig. 1; Additional file 1: Figure S1 and Additional file 1: Figure S2a). For each sample, we created the detailed description by extracting the ancestry descriptor utilized by the author in the relevant publication. To generate the controlled description, we selected, from a limited list of terms (Table 1), the category noted by the author or, if not stated, the category that best correlates with the detailed description for the same sample. For example, we selected the category “East Asian” for detailed descriptions containing the descriptor “Han Chinese”.

We relied heavily on data stated by authors in the GWAS publication, giving precedence to information inferred using genomic methods, such as principal component analysis (PCA; see Additional file 1: Box S1 for a list of methods commonly used to ascertain ancestry). In some cases, we considered other sources, but only when the information provided by authors was limited or ambiguous. We consulted peer-reviewed population genetics publications to obtain additional information on lesser-known groups that were not adequately characterized by authors or when samples were described using ethno-cultural terms (for example, “Punjabi Sikh”). When the only information provided in the publication was the location of recruitment, we consulted The United Nations M49 Standard of Geographic Regions [12] and The World Factbook [13]. The latter is a regularly updated compendium of worldwide demographic data, covering all countries and territories of the world. Additional file 2: Table S1 provides a list of countries of recruitment in the Catalog, together with the sources that were consulted and the inferred categories.

In rare instances, the ancestry information provided by authors was not detailed enough to allow the resolution of samples into ancestrally distinct sets. For these samples, we created complex, multi-ancestry detailed descriptions and selected multiple categories (for example, Catalog entry for Jiang R et al. [14, 15]). For admixed samples, we selected either one of the categories that includes individuals with well-defined admixture (“African American or Afro-Caribbean” and “Hispanic or Latin American”) or the category “other admixed ancestry”. We also captured additional information to describe the ancestral backgrounds that contribute to the admixture. No ancestry-informative detailed descriptions were generated in the absence of ancestry or recruitment data; for those samples, the category “Not Reported” was selected.

Where possible we also curated country of recruitment (Fig. 1; Additional file 1: Figure S2b) and country of origin as this provides additional and complementary demographic information. Country of origin was extracted when the country of origin of the study participant’s grandparents was stated or when the genealogy of the sample could be traced to a particular country.

The detailed extraction guidelines utilized by Catalog curators are included in Additional file 1: Supplementary Methods. A full list of Catalog detailed descriptions and categories is provided in Additional file 3: Table S2. Examples that illustrate application to specific samples can be found in Additional file 4: Table S3. All curated ancestry data are available from the GWAS Catalog website [4] (Fig. 1) and via download [16].

### Improving data analysis and assessing diversity

Taking advantage of this fully curated and well described data set, we performed a new and enhanced survey of the ancestral background of Catalog samples. Similar analyses have been previously performed [1, 2]. However, these have focused exclusively on the detailed descriptions, which are more complex and heterogeneous. Our analysis uses, for the first time, categories and goes beyond individuals to studies, associations, traits, and change over time.

**Fig. 2:**
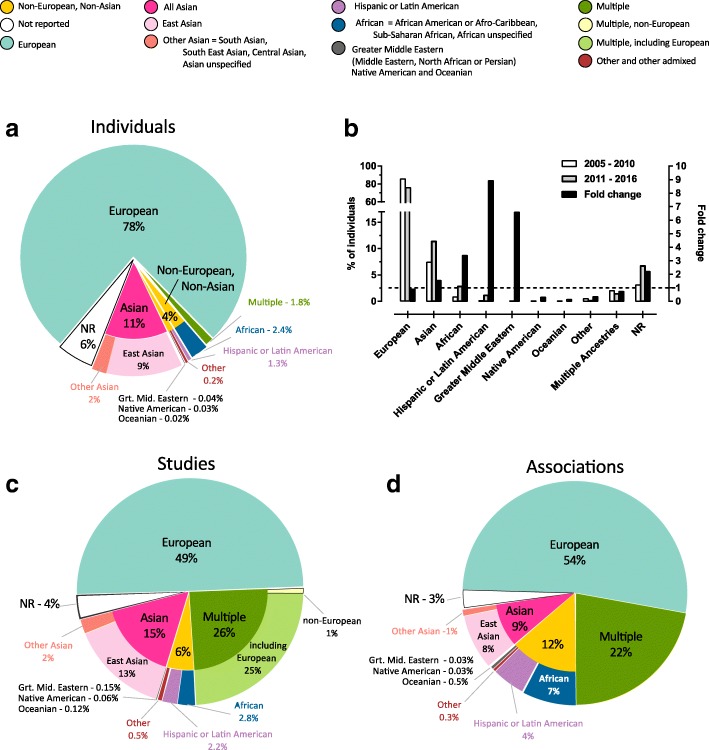
Ancestry category distribution in the GWAS Catalog. This figure summarizes the distribution of ancestry categories in percentages, of individuals (N = 110,291,046; **a**), individuals over time (N = 110,291,046; **b**), studies (N = 4,655; **c**), and associations (N = 60,970; **d**). The largest category in all panels is European (*aqua*). At the level of individuals (**a**), the largest non-European category is Asian (*bright pink*), with East Asian (*light pink*) accounting for the majority. Non-European, Non-Asian categories together (*yellow*) comprise 4 % of individuals, and for 6 % (*white*) of samples no ancestry category could be specified. **b** The distribution of individuals in percentages, included in the 915 studies published between 2005 and 2010 compared to the distribution of individuals included in the 2905 studies published between 2011 and 2016. **d** The disproportionate contribution of associations from African (*blue*) and Hispanic/Latin American (*purple*) categories, when compared to the percentage of individuals (**a**, *blue*, *purple*, respectively) and studies (**b**, *blue*, *purple*, respectively)

As previously reported [2], we found that the majority (78 %) of individuals in the Catalog are exclusively of European ancestry (Fig. 2a), followed by individuals of East Asian descent (9 %). The disproportionate focus on Europeans was more prevalent in the earlier years of the Catalog (86 % of individuals in studies published between 2005 and 2010; 76 % between 2011 and 2016), with a notable increase in African (0.8 to 2.8 %, 3.5-fold increase), Hispanic or Latin American (0.1 to 1.2 %, ninefold increase) and Middle Eastern (0.01 to 0.08 %, sevenfold increase) samples in the last 5 years (Fig. 2b). Despite this trend, however, these non-European, non-Asian groups combined account for less than 4 % of the Catalog’s individuals. We observed a similar result when analyzing GWA studies. Almost 50 % of all studies exclusively analyze European ancestry individuals, and an additional 25 % of studies analyze multiple ancestries, including individuals of European descent (Fig. 2c).

Interestingly, when we focused on the number of associations contributed by each category, we noted a disparity with respect to the distribution observed when analyzing individuals (Fig. 2d). This was particularly pronounced for studies including African or Hispanic or Latin American samples, many of which are African-admixed [17]. African ancestries comprise 2.4 % of individuals but contribute 7 % of associations. Similarly, only 1.3 % of individuals in the Catalog are Hispanic or Latin American, yet they contribute 4.3 % of associations. The opposite effect was seen in Europeans, with 78 % of individuals yet only 54 % of associations.

Our ability to observe this disproportionate yield of associations is directly correlated with the use of categories in our analysis. The benefits of our framework, however, extend beyond assessing diversity to the pursuit of scientific questions. Using our categories, we were able to identify diseases or traits that have been analyzed in a large number of ancestral backgrounds and use this information to search for loci and variants that generalize across ancestries as well as loci or variants that may have ancestry-specific impact. For example, we found that type 2 diabetes has been analyzed in multiple ancestral backgrounds (29 distinct detailed descriptions and 12 categories across 52 studies and 610 associations). We then reviewed all loci associated with this disease and found that some (for example, 10q25.2) appear to generalize across many ancestral groups and others seem limited to a small number (for example, 4p16.3 primarily in Asians). The assignment of our categories to the 1000 Genomes and HapMap project populations enables a more focused review of ancestry-specific LD and allele frequency information for these loci, and this, in turn, can inform study designs aimed at fine mapping and the identification of causal variants. This process also allows the identification of clear gaps in the data, such as particular ancestral backgrounds that have yet to be analyzed.

### Application beyond the GWAS Catalog

To encourage widespread adoption of the framework, we here pursue three approaches.

#### Recommendations for authors

Curation of GWAS publications revealed inconsistent and ambiguous reporting of ancestry data, with a significant percentage of studies (~ 4 %) not reporting any relevant information at all. Therefore, we provide a set of specific recommendations for authors, summarized in (Table 2), that require minimal additional burden, and, if implemented, will improve the quality of reporting and have a positive impact on the interpretation of published results, data re-use, and reproducibility.

**Table 2:** Recommendations for authors reporting ancestry data in publications. These recommendations were generated by expert curators following a detailed review of the over 3200 GWAS publications included in the Catalog

1. Provide detailed information for each distinct group of samples, a. Ancestry descriptors should be as granular as possible (e.g., Yoruban instead of Sub-Saharan African, Japanese instead of Asian). b. Avoid using country or citizenship as a substitute for ancestry. c. Avoid using geographic descriptors that are part of a cohort name as a substitute for ancestry (e.g., TwinsUK cannot be assumed to be European ancestry). d. If a population self-identifies using sociocultural descriptors, clearly provide information about the underlying genetics or genealogy (e.g., Old Order Amish individuals of European descent) e. If samples were derived from an isolated or founder population with limited genetic heterogeneity, clearly state the genetic ancestry within which this sub-population falls. f. Every effort should be made to explicitly note whether the population is admixed and the ancestral backgrounds that contribute to admixture. g. If available, genetic genealogy or ancestry of grandparents or parents should be included. 2. Report the method used to determine the ancestry of participants (for example, self-reported, inferred by genomic methods, or a combination of both) a. Where possible, use genomic methods to confirm self-reported ancestry or to infer the ancestry of samples. b. If inferred, indicate the analytical procedure utilized. See Additional file 1: Box S1 for a description of commonly used methods. 3. Assign an ancestry category for each distinct group of samples. See Table 1 for a list of ancestry categories. Refer to Additional file 3: Table S2 for a list of descriptors in use in the Catalog with their category assignments. 4. Provide the sample size for each distinct group of samples included in the analysis. 5. Provide country of recruitment. 6. If ancestry information is not available due to confidentiality, or any other concerns, note this in the publication.	

We recommend that authors make every effort to generate a detailed description for each distinct set of individuals included in their studies. Authors should also note a corresponding category by assessing whether the genetic diversity of each distinct set is representative of one of the known populations listed and defined in Table 1. Where possible, we recommend authors assess the ancestry using genomic methods (Additional file 1: Box S1), as this will aid the classification process. If authors have no knowledge about the ancestry of the participants, are not able to infer it, or cannot share it due to confidentiality concerns, we suggest noting this explicitly in the publication.

In general, terms that pertain to an individual’s ethno-cultural background should be avoided, unless this provides additional information regarding the genealogy of the samples. In such cases a descriptor that accurately reflects the underlying genetics should also be provided. For example, when describing “Punjabi Sikh” participants, authors should also describe the samples as “South Asian” or “Punjabi Sikh South Asian” rather than simply “Punjabi Sikh” or “Sikh”.

Particular care should be taken to note if a sample derives from a founder or genetically isolated population. Given their homogeneity and reduced genetic variation, these populations are especially well-suited for GWAS [18] and are increasingly used as sample sources. When describing isolates, the broader genetic background within which the population clusters should also be indicated. For example, Old Order Amish participants should be described as “Old Order Amish population isolate individuals of European descent”, for example.

While describing admixed populations can be challenging due to varying levels of admixture, every effort should be made to explicitly note whether the sample is admixed and the ancestral backgrounds that contribute to admixture. For example “Hispanics/Latinos are ethnically heterogeneous, with admixture of European, West African, and Amerindian ancestral populations”, as stated in Hodonsky et al. [19].

### Ancestry-specific ontology

To facilitate application to bioinformatics resources, we developed and released an ancestry-specific ontology based on our curated GWAS Catalog descriptions. We have defined terms, identified synonyms, and established hierarchical relationships between all curated terms and categories. The use of this ontology in any search interface will enable users to perform more powerful and precise ancestry-related queries [20]. We aim to integrate it into the GWAS Catalog website in the near future. The ancestry ontology [21] can be browsed and downloaded (manuscript in preparation).

### Application to reference populations

The HapMap [7] and 1000 Genomes [6] projects have collated a number of widely used reference populations and delivered a comprehensive survey of human genetic variation. The application of our framework to these populations, therefore, provides huge integration potential, especially with any samples described using these references in PCA and other analyses. For all HapMap and 1000 Genomes phase 3 populations, we assigned ancestry category, country of recruitment, country of origin, and a detailed description, if provided by each project (Additional file 5: Table S4).

## Discussion

### Summary

In this report, we describe a framework for the standardized representation of ancestry data from genomics studies. Our method provides structure to unstructured data, enabling robust searching across large datasets and integration across resources.

### Limitations of the framework

Despite the successful application of our method to GWAS Catalog samples and to commonly used reference populations, there are challenges. We are aware of the sensitivities surrounding the topics of ancestry, race and ethnicity, and the difficulties that arise when trying to classify the global human population. Due to evolution and patterns of migration, the ancestry of a particular population is complex. However, it is both possible and useful to generate standardized terminology and to classify individuals into informative groupings. Reference populations or ancestry informative markers [22] that allow populations to be distinguished have been characterized, and methods have been developed to adjust for population stratification and separate samples into clusters. Practically, the classification of samples into categories facilitates data integration and allows robust searches, which is an essential component of databases such as the GWAS Catalog. Also, as we demonstrate in our survey of Catalog ancestry data, the use of categories can greatly facilitate further analyses by, for example, reducing the complexity of data sets.

We recognize that as more cohorts from diverse populations are characterized, there might arise a need to create additional categories or sub-categories. Also, it is likely that admixture will increase in the future, due to migration, for example, resulting in samples that could be described using multiple categories. The classification of admixed samples is particularly challenging. The degree and type of admixture may vary within the population, and the accuracy of classification requires well-defined reference samples, which are lacking for some groups. In an effort to address this, we have created categories to represent admixed groups that are known (for example, “Hispanic or Latin American”) and emerging (for example, “Other admixed ancestries”). We have also included, and recommended inclusion of, information regarding the populations that contribute to admixture. We note that since the vast majority of admixed Catalog samples can be classified as either “Hispanic or Latin American” or “African American or Afro-Caribbean”, we felt it was sufficient to create one category to include all other forms of admixture. However, we recognize that as the community moves towards increased characterization of these groups, using genomic methods, for instance, our admixed categories are likely to become more precise and granular over time.

### Assessing diversity in genomics

Several reports have been published urging the scientific community to ensure that individuals from all backgrounds benefit from advances in the field of genomics [1, 2]. However, this requires the establishment of metrics and proper tracking of ancestry data over time. As evidenced by our new survey of ancestral backgrounds, we believe the widespread implementation of our framework, especially the use of standardized language and categories, can yield important benefits in this area.

There are, however, limitations to the use of categories to track diversity in the Catalog. Considering that some cohorts have been included in numerous studies, some individuals are represented multiple times. The impact of this is the skewing of results towards commonly used or publicly available cohorts, which are likely of European or Asian descent. Also, associations identified in multi-ancestry studies, for example, “trans-ethnic” discoveries or multi-ethnic replications, could not be described using one category, resulting in a disproportionate number of “multiple” ancestry associations (1.8 % individuals, 22 % associations; Fig. 2d). This may contribute to the reduced proportion of associations attributed to European populations, since the vast majority of “multiple” ancestry studies include Europeans (Fig. 2c).

While the general bias towards inclusion of European ancestry samples in GWA studies has been previously reported, the disparity in the yield of associations derived from African and Hispanic or Latin American populations is a novel observation. We suggest that the higher degree of genetic diversity and reduced linkage disequilibrium (LD) in African [23] and African-admixed populations offers an explanation for this result. Shorter LD blocks in African populations facilitate the separation of nearby but independent signals in a way that is more challenging in populations with shorter LD blocks, such as Europeans and Asians. Also, as the number of individuals from African and Hispanic or Latin American populations has grown over the years, the power to discover additional disease-associated variants by leveraging the increased genetic diversity in these populations has improved.

The benefit of including diverse populations has been articulated, and extends throughout the translational research spectrum, from GWAS discovery efforts to genomic medicine. For example, studies including multiple populations may aid in fine mapping of existing signals or in identifying population-specific functional variation [6, 24]. Also, variant interpretation for genomic medicine in ancestrally diverse or admixed populations relies on the availability of non-European variation information, with potentially serious clinical consequences if such data are not available [25]. While we are encouraged by the trend we have seen in recent years towards increased diversity, we note that there are still very clear gaps as some groups continue to be underserved or ignored. We strongly urge the scientific community to expand their efforts to assemble and analyze cohorts, including especially underrepresented communities.

Human genomics studies, including GWAS, have been enormously successful [3, 5, 26]. However, the ability to properly interpret and query the generalizability of results across populations requires clarity about the ancestry of samples. Therefore, we have provided a framework for the standardized representation of ancestry. We believe widespread adoption will enable the scientific community to investigate the generalizability of genotype–trait associations across diverse populations, to identify associations more prevalent in specific ancestries, to identify novel variants with clinical implications, and to help pinpoint causative variants, thus increasing our understanding of common diseases.

## Methods

### GWAS Catalog data curation

GWAS Catalog eligibility criteria and general curation methods can be found on the GWAS Catalog website. Curation of ancestry data from the literature was performed according to the Ancestry Extraction Guidelines outlined in Additional file 1: Supplementary Methods.

### GWAS Catalog ancestry analysis

To determine the distribution of individuals, associations and traits by ancestry category, we first downloaded all Catalog data in tabular form [16]. All data included in these analyses were curated from GWA studies published between 2005 and the end of 2016, with a release date of July 18 2017. The data can be found on the Catalog’s FTP site [27] (gwas-catalog-associations_ontology-annotated.tsv, gwas-catalog-ancestry.tsv, gwas-catalog-studies_ontology-associated.tsv, and gwas-efo-trait-mappings.tsv).

### 1000 Genomes and HapMap Project population ancestry assignment

Information describing the 1000 Genomes [6] phase 3 and HapMap Project [7] phase 3 populations was taken from the Coriell Institute website [28]. Ancestry information, including ancestry category, country of recruitment, country of origin, and additional information, was assigned to each population following the GWAS Catalog Ancestry Extraction Guidelines mentioned above.

## Additional files

Additional file 1: **Figure S1.** Detailed sample description displayed in the internal GWAS Catalog curation interface. **Figure S2. a** Structured ancestry and recruitment information displayed in the internal GWAS Catalog curation interface. **b** GWAS Catalog ancestry and recruitment data entry page of internal curation interface. **Supplementary Box 1.** Genomic methods of ancestry determination. **Figure S3.** Distribution of studies by ancestry category focused on Catalog traits with highest number of studies in the Catalog. **Figure S4.** Methods of ancestry ascertainment used in a subset of publications included in the GWAS Catalog. **Supplementary References**. (DOCX 893 kb)

Additional file 2: **Table S1.** GWAS Catalog countries of recruitment for which no ancestry information was provided. (XLSX 77 kb)

Additional file 3: **Table S2.** GWAS Catalog detailed descriptions with ancestry category assignments. (XLSX 77 kb)

Additional file 4: **Table S3.** Specific examples to illustrate the application of the framework to the GWAS Catalog. (XLSX 70 kb)

Additional file 5: **Table S4.** HapMap Project and 1000 Genomes Project populations with assigned ancestry category. (XLSX 27 kb)
