# Supplementary material for: A standardized framework for representation of ancestry data in genomics studies, with application to the NHGRI-EBI GWAS Catalog
Source: Genome Biol. 2018 Feb 15;19:21. doi: 10.1186/s13059-018-1396-2 (PMC5815218; doi:10.1186/s13059-018-1396-2)
Supplement: Supplementary file 1 — Figure S1. Detailed sample description displayed in the internal GWAS Catalog curation interface. Figure S2. a Structured ancestry and recruitment information displayed in the internal GWAS Catalog curation interface. b GWAS Catalog ancestry and recruitment data entry page of internal curation interface. Supplementary Box 1. Genomic methods of ancestry determination. Figure S3. Distribution of studies by ancestry category focused on Catalog traits with highest number of studies in the Catalog. Figure S4. Methods of ancestry ascertainment used in a subset of publications included in the GWAS Catalog. Supplementary References. (DOCX 893 kb) [file 13059_2018_1396_MOESM1_ESM.docx]

**SUPPLEMENTARY INFORMATION**

**A standardized framework for representation of ancestry data in genomics studies, with application to the NHGRI-EBI GWAS Catalog**

Joannella Morales^1*^, Danielle Welter^1^, Emily H. Bowler^1^, Maria Cerezo^1^, Laura W. Harris^1^, Aoife C. McMahon^1^, Peggy Hall^2^, Heather A. Junkins^2^, Annalisa Milano^1^, Emma Hastings^1^, Cinzia Malangone^1^, Annalisa Buniello^1^, Tony Burdett^1^, Paul Flicek^1^, Helen Parkinson^1^, Fiona Cunningham^1^, Lucia A. Hindorff^2^, Jacqueline A. L. MacArthur^1*^

*^1^European Molecular Biology Laboratory, European Bioinformatics Institute, Wellcome Genome Campus, Hinxton, Cambridge, CB10 1SD, UK*

*^2^Division of Genomic Medicine, National Human Genome Research Institute, National Institutes of Health, Bethesda, MD 20892, USA*

*Joannella Morales and Jacqueline MacArthur are corresponding authors.

Lucia A. Hindorff and Jacqueline A.L. MacArthur share joint last authorship of this manuscript.

**Supplementary Methods**

GWAS Catalog Ancestry Extraction Guidelines

Sample ancestry information is captured in the GWAS Catalog in two distinct forms; a “detailed” sample description (Supplementary Figure 1), and a “structured” description (Supplementary Figure 2) that contains the ancestry category and country information. Detailed descriptions aim to capture accurate, informative and comprehensive information regarding the ancestry or genealogy of the samples. Ancestry categories are used to establish hierarchical relationships between groups and populations, thus facilitating data representation, integration and database searches. The framework also allows for country information to be recorded providing additional detail on sample demographics. All sample ancestry information is based on information provided by the author in the curated paper or information contained or referred to within the GWAS Catalog Ancestry Extraction Guidelines and referenced materials.

Detailed sample description

Detailed descriptions for the initial and replication stages of the GWAS are entered separately as “Initial Sample Description” and “Replication Sample Description”. These provide summary descriptions of the samples analyzed in each stage. The initial sample description includes all samples in which all genome wide SNPs passing quality control (QC) are analyzed. The replication sample description comprises the samples in which a selected number of SNPs from the GWA stage are followed up. The sample description reflects the study design being extracted, with samples split into “cases” and “controls” if appropriate. For quantitative analyses or when no case/control sample numbers are provided samples are described as “individuals”. Samples may also be described according to their family structure, for example “trios” or “families”, or using a more specific sample description, e.g. “children”, if this is important for the study design. Only information from the paper (or these extraction procedures and referenced materials) is included in the Catalog; for example, in a set of related papers published together, only information from each paper is included in the respective Catalog entry (even if other papers are referenced).

Sample size

- The post-QC sample size is reported where available. If this is unavailable then pre-QC sample size is reported.
- When describing family units, for example trios or sib-pairs, the number of units is included in the detailed description, whereas the number of individuals is included in the structured data. For example, the detailed description for the GWAS published in Hancock DB et al., 2009[1] includes the number of trios - “492 Mexican ancestry trios”. The actual number of individuals (1,476) is included in the structured ancestry data.
- Where no sample number is provided in the publication a citation may be included for the sample cohort if provided by the author. For example, the detailed description for the GWAS published in Lawrance-Owen et al., 2013[2] states “(see Medland, 2010)”.
- If the authors do not carry out a replication step, “NA” is entered.
- “Up to” is used as a qualifier before the sample size when a single Catalog entry includes multiple GWA analyses on different sized subsets of the same cohort.

Ancestry description

- Catalog detailed descriptions include the most detailed ancestry descriptor provided by the author, e.g. “Han Chinese”, “Black”, “Jewish”, “Thai”, “Colored African”.
- Samples described in a publication as “White” or “Caucasian” are described in the detailed description as European.
- If multiple populations of the same ancestry category are included in a study, the samples are combined under the ancestry category descriptor (Table 1). For example, for a study that includes 100 Japanese ancestry individuals and 200 Korean ancestry individuals, the detailed description states "300 East Asian ancestry individuals". When multiple sample populations are combined in this way the descriptions provided by the author for each sample population are included in the “Additional information” field in the structured ancestry data (Supplementary Figure 2, GWAS Catalog study entry GCST004735 [3]).
- All ancestry descriptors currently used in the Catalog, along with the assigned ancestry category, are shown in Supplementary Table 1.
- It is assumed that a descriptor, e.g. “Dutch”, refers to ancestry and not citizenship or country of recruitment unless information is provided to contradict this. For example, if the author states “100 Dutch cases”, European ancestry is inferred, rather than recruitment in The Netherlands.

Founder/genetically isolated populations

- A founder population or genetic isolate has genetic homogeneity or limited genetic variation within the population. Language that would classify the population as a “founder population” or “genetic isolate” includes:
  - Description of the population as descended from founder individuals or as having isolated population genealogy
  - Detailed/well recorded family tree with no/limited admixture
  - Description of the population experiencing founder effects
- For example: “The Orkney Complex Disease Study (ORCADES) is an ongoing family-based, cross-sectional study in the isolated Scottish archipelago of Orkney. Genetic diversity in this population is decreased compared to Mainland Scotland, consistent with the high levels of endogamy historically”.
- A description of geographical or political isolation is not enough to identify the population as a genetic isolate. Reference must be made to genealogy or population genetics.
- Each founder/genetic isolate descriptor is decided on a case by case basis, using the authors’ language and description of the sample from the paper being curated, followed by the term “founder/genetic isolate”. For example, the Sardinian population isolate from Sardinia, Italy is listed as “Sardinian (founder/genetic isolate)”.

Admixed Populations

- Populations with ancestry from more than one distinct parental population are considered to be admixed.
- Currently, the vast majority of Catalog samples with recent admixture can be categorized as “African American or Afro-Caribbean” or “Hispanic or Latin American”. When describing these admixed samples, Curators should follow the guidelines described above.
- When describing samples with recent admixture distinct from these populations, the detailed descriptor is decided on a case by case basis, using the authors’ language from the paper being curated.

Structured ancestry and recruitment information

The structured data for each distinct sample population includes the ancestry category from Table 1, and country of origin and recruitment information provided by the author.

Ancestry Category

- Each detailed descriptor is mapped to an ancestry category (Table 1) and the samples are combined if they belong to the same category.
- The individuals included in each category are expected to have genetic variation representative of, or known relatedness to, the population in these regions, excluding recent migrations.
- The mappings of detailed descriptions to the ancestry categories should be carefully considered for each study. If the author reports that ancestry is both "genetically assessed" and "self-reported", then the sample ancestry in the Catalog is reported according to the genetically assessed methods.
- All ancestry descriptors currently used in the Catalog, along with the assigned ancestry category are shown in Supplementary Table 1. Ancestry descriptors not already included in the Catalog are assigned an ancestry category on a case by case basis. The decision is based on information provided by the author (taking into account ancestry ascertainment methods, as mentioned above) and, where necessary, information provided by The World Factbook[4], the geographical region and additional published resources.
- Founder populations are assigned the ancestral category as described by the author. If no ancestral category is given these are assigned the ancestral category “Other” (see Supplementary Table 1).
- The following ancestry categories are used if the individuals cannot be classified in any of the broad categories:
  - “Not Reported” - “NR” is entered if no ancestry is reported in the paper and any country of recruitment provided cannot be used to infer the ancestry. “NR” is also used if the author classifies the samples as “other ancestry” when listing several ancestral categories.
  - “Other” - “other” is entered if an ancestry descriptor is provided by the author but insufficient information is given to allow us to assign it to one of the categories, e.g. “Russian”.
  - “Other admixed ancestry” – “other admixed ancestry” is entered if the author has stated that the samples are admixed but the admixture is other than the known admixture represented in the categories “African American or Afro-Caribbean” and “Hispanic or Latin American” (for example, GWAS Catalog entry for Xia K et al.[5]).
- When the sample includes multiple ancestral groups and the author does not provide the sample size for each of these, the total sample size is given and multiple categories selected. For example, if the analysis included 200 French, Mexican or Chinese individuals, “European, Hispanic/Latin American and East Asian” would be selected as categories for the 200 individuals.
- “Asian unspecified” or “African unspecified” is used only if no additional information is provided that would enable curators to assign a more specific ancestry category. For example, “200 Asian individuals recruited in Malaysia, Singapore, and the U.S.” is listed as “Asian unspecified”.
- If the ancestry information provided by the author contradicts the ancestry inferred by geographical region or country of recruitment, then the ancestry provided by the author is used. For example, “200 Caucasian individuals recruited in Mexico” is described as European, and not as Hispanic or Latin American.

Country of origin

- Country of origin is entered if the author provides details of the country of origin of an individual’s grandparents. For example, in the Catalog entry for [Melén](https://www.ncbi.nlm.nih.gov/pubmed/?term=Mel%C3%A9n%20E%5BAuthor%5D&cauthor=true&cauthor_uid=23517042) et al., 2013[6], “Of these 2,714 children, 616 (22.7%) unrelated children had high probability of having at least 6 great-grandparents born in the Central Valley of Costa Rica”.
- Country of origin is also entered if there is evidence of known genealogy associated with a country of origin, e.g. “knowledge of Icelandic genealogy” (see Stacey, 2011[7]) has been used to justify assigning country of origin.
- “NR” is entered when no country of origin is provided.

Country of recruitment

- Country of recruitment of the sample is entered if stated by the author. Country of recruitment is not inferred from an ancestry identifier e.g. “100 Thai cases” does not necessarily mean that country of recruitment is “Thailand”.
- Country of recruitment is not assumed from a cohort or Biobank name (e.g. “Twins UK”, “Framingham Heart Study”); the specific location of recruitment of the samples must have been mentioned. An exception to this is if the samples are referred to as being recruited within a National Health Scheme or similar.
- When the descriptor "African American", “European American”, “Mexican American” or “American Indian” is provided, the U.S. is entered as the country of recruitment.
- “NR” is entered when no country of recruitment is provided for a given broad ancestral category.

Additional description

- All ancestry descriptors provided by the author are entered in the “Additional description” under the “Ancestry category” to which they have been mapped (this applies to GWAS Catalog studies from January 2016 onwards).
- When describing admixed samples, if provided by the author, the distinct ancestral backgrounds that contribute to admixture are entered in the “Additional description” under the “Other admixed ancestry” category.

Where no ancestry information is provided

- When no ancestry descriptor is used by the author but a country of recruitment is stated, curators use additional published materials to infer the structured ancestry category. This includes relevant ancestry publications, and population information provided by The World Factbook[4].
- The World Factbook is a reference resource, produced by the U.S. Central Intelligence Agency, which provides information about the demographics, geography, communications, government, economy, and military of countries. This may include information on the ancestry of groups that make up the population of a country**.** This information is used to infer the ancestry of a study sample when the country of recruitment of the samples is the only information provided. Ancestry is only inferred for countries with > 90% of the same ancestry according to The World Factbook. Supplementary Table 3 provides a list of countries for which The World Factbook and other sources, also listed therein, were consulted in order to select a Catalog category.
- Ancestry is not assumed for countries with < 90% of the same ancestry; in this case “NR” is entered.
- While The World Factbook is used for the majority of countries, other resources are consulted for a number of countries with a high proportion of admixture. For example, scientific papers have been consulted to provide ancestry information on countries in Latin America and the Caribbean (Supplementary Table 3)
- The ancestry identifiers “Other” and “Other admixed ancestry” are used for countries that span more than one broad geographic area and therefore are likely to have a diverse population or heavily admixed population. For example, if a study includes 200 individuals recruited in Russia, and no ancestry data is provided in the publication, the detailed description states “200 other ancestry individuals” and the category “Other” is included in the structured data.
- Country of recruitment is only entered when stated by the author and never assumed based on ancestry information. For example, if the author states “Chinese ancestry” we do not assume the country of recruitment is China.
- When authors do not report an ancestry and this cannot be inferred from the country of recruitment, the samples are described as “# individuals” or “# cases, # controls”. For example, 100 individuals recruited from a mixed population such as in the United States, will be described as “100 individuals”.

Examples

- See Supplementary Table 2 for further examples of how these extraction guidelines are implemented in the GWAS Catalog.

Analysis of ancestry assessment methods in a subset of the GWAS Catalog

To perform a general survey of the methods utilized by authors to assess ancestry, we selected the first 100 publications included in the Catalog (approximately covering the period between March 2005 to January 2008), and for comparison, the first 100 publications from 2016. For each publication, the method was reviewed and classified into one of the following: 1. Self-reported, 2. Genetically assessed, 3. Ancestry stated without method, 4. Inferred from limited ancestry-related information (e.g. country information), 5. No ancestry information reported and 6. Mixed method (when a combination of methods was utilized to describe the study samples). Publications classified as “Genetically assessed” includes those where the author had clearly identified the genetic ancestry or admixture of the population, for example by using methods such as those described in Supplementary Box 1. It also includes those that confirmed self-reported information or defined samples based on self-reports but then excluded genetic outliers. Publications where no ancestry was stated, but curators inferred an ancestry based on country information are included in the fourth classification. In many cases authors used a statistical method to assess or control for ancestry or population stratification, without assigning individuals to a particular category, for example using a continuous axis of genetic variation from PCA to compute the association statistic. However, since this did not add any information that curators could use to assign a population ancestry to the study, it was not included under category 2.

**Supplementary Box 1** - Genomic methods of ancestry determination

| Approach | Representative Method/Software | Description |
| --- | --- | --- |
| Mixture model | STRUCTURE, ADMIXTURE | STRUCTURE[8] analyses differences in the distribution of genetic variants amongst populations with a Bayesian iterative algorithm by placing samples into groups whose members share similar patterns of variation, either allowing for admixture or not. ADMIXTURE[9] is a software tool for maximum likelihood estimation of individual ancestries from multilocus SNP genotype datasets designed for efficiency in large GWAS datasets. It uses the same statistical model as STRUCTURE but using a different optimization algorithm. |
| Principal components analysis | EIGENSTRAT | PCA is a multivariate method used to infer continuous axes of genetic variation (eigenvectors) that maximize the variance explained in a small number of dimensions, whilst describing as much of the variability between individuals as possible.  GWAS data can be analysed alone or combined with that from reference samples at the same SNPs. Populations can then be identified and outliers removed if necessary. PCA can also be used to correct for population stratification by creating sets of matched cases and controls; alternatively this information can be included in ancestry-adjusted association analyses such as multiple regression[10]. |
| Multi-dimensional scaling | PLINK | Multi-dimensional scaling (MDS), a related multivariate statistical technique, can also be used to estimate axes of genetic variation. The MDS method detects meaningful underlying dimensions that explain observed genetic distance, e.g., pairwise identity-by-state (IBS) distance, among individuals rather than Euclidean distance in PCA[11]. |
| Mixed effects models | EMMAX | These methods effectively control for stratification within a population and are a popular alternative to PCA for this purpose (reflecting data structure not ancestry per se). The mixed effects model method models population structure and cryptic relatedness as random effects, while can taking into account fixed effects, such as age and gender[12]. |

**Supplementary Tables** (Separate Excel spreadsheets)

1. Supplementary Table 1. GWAS Catalog countries of recruitment for which no ancestry information was provided.
2. Supplementary Table 2. Detailed descriptors currently used in the Catalog with assigned ancestry category.
3. Supplementary Table 3. Specific examples to illustrate the application of the framework to the GWAS Catalog.
4. Supplementary Table 4. HapMap Project and 1000 Genomes Project phase 3 populations with assigned ancestry categories.

**Supplementary Figures**

1. Supplementary Figure 1. Detailed sample description displayed in the internal GWAS Catalog curation interface.


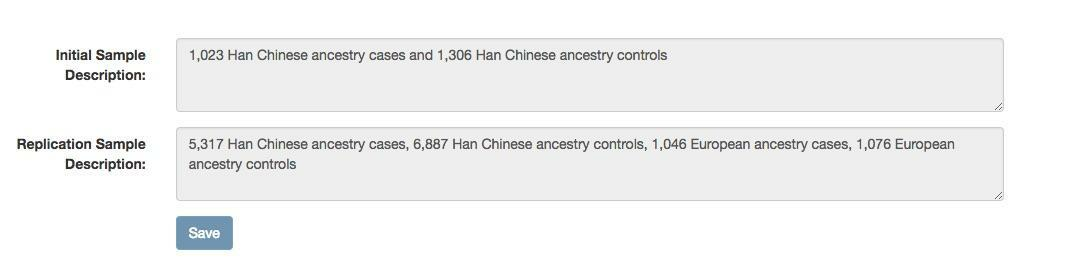


1. Supplementary Figure 2a. Structured ancestry and recruitment information displayed in the internal GWAS Catalog curation interface.


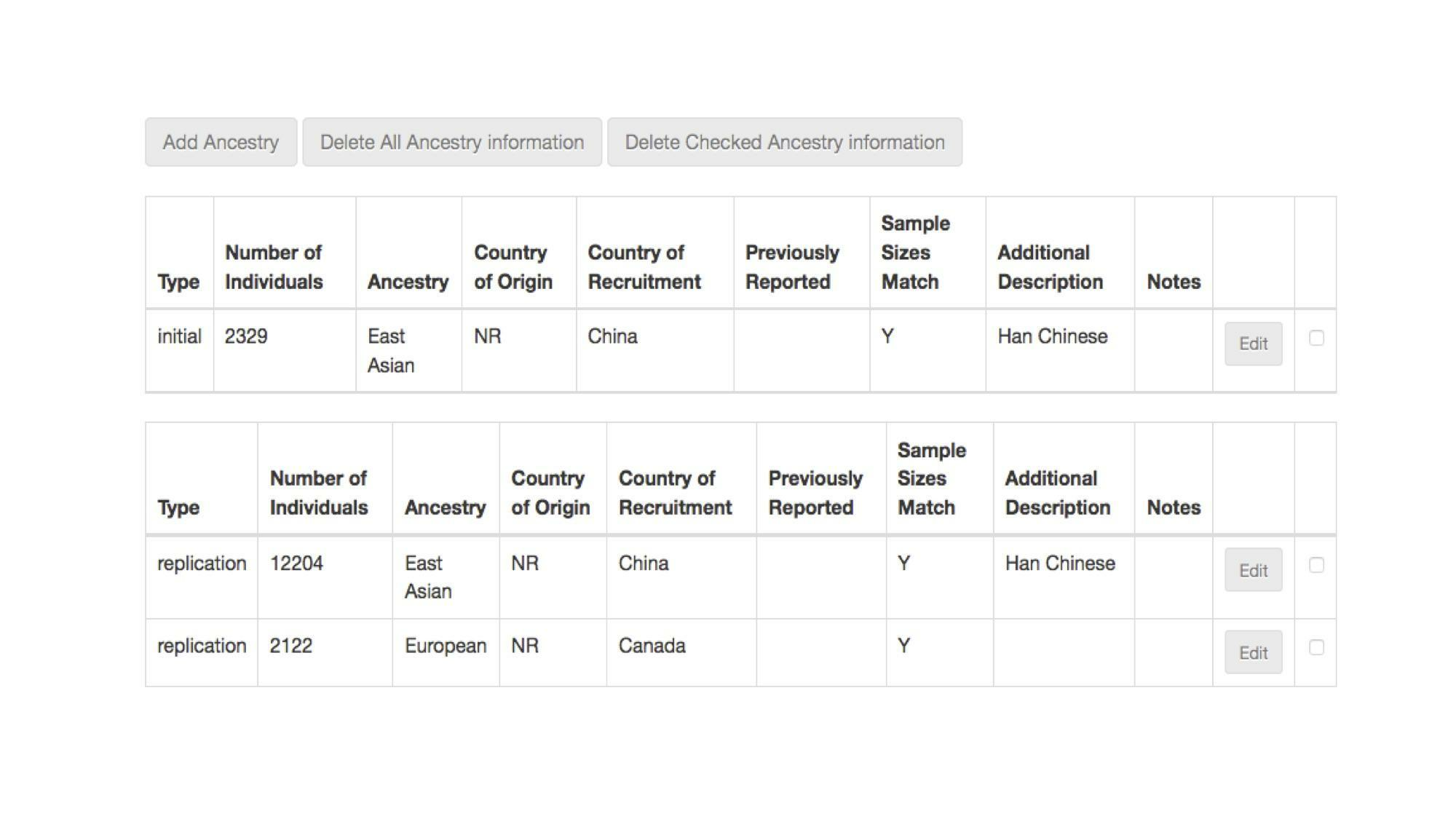


1. Supplementary Figure 2b. GWAS Catalog ancestry and recruitment data entry page of internal curation interface.


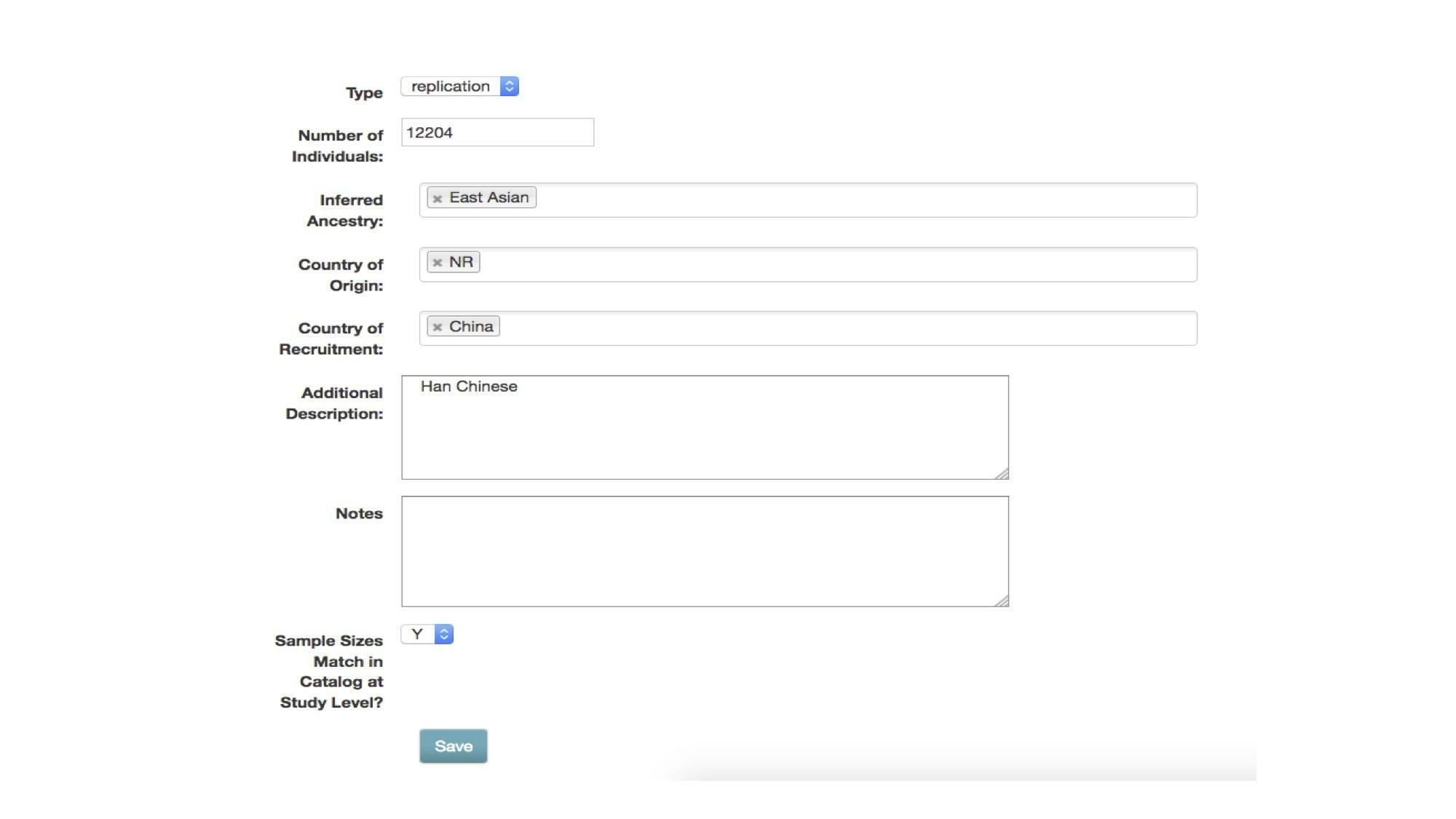


4. Supplementary Figure 3. Distribution of studies by ancestry category focused on Catalog traits with highest number of studies in the Catalog. This figure displays the top ten most common traits in the GWAS Catalog and the percentage of individuals from each ancestry category. The traits with the greatest representation of ancestrally diverse individuals, based on number of ancestry categories represented, are anthropometric traits (BMI and Body Height) and common diseases (Type 2 Diabetes and Heart Disease). The Catalog-wide bias towards inclusion of European ancestry individuals is also observed in all traits analyzed (aqua and green).

5. Supplementary Figure 4. Methods of ancestry ascertainment used in a subset of publications included in the GWAS Catalog. This figure displays the methods of ancestry ascertainment found in the first 100 publications included in the Catalog (approximately covering the period between March 2005 to January 2008) and the first 100 publications from 2016. For each publication, the method was assessed and classified into one of the following: 1. Self-reported, 2. Genetically assessed, 3. Ancestry stated without method, 4. Inferred from country information and 5. No ancestry information reported. When more than one method was utilized, publications were classified as 6. Multiple methods.


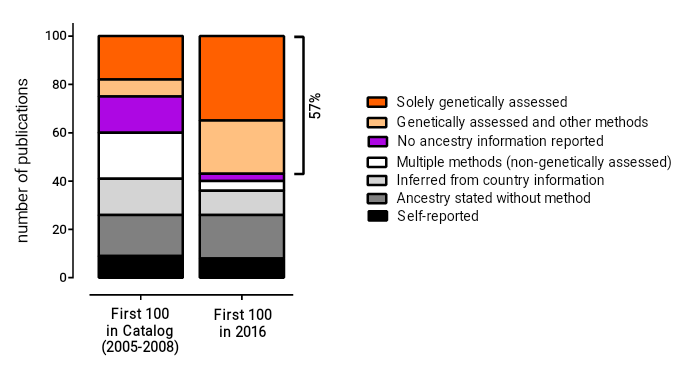


**Supplementary References**

1. Hancock DB, Romieu I, Shi M, Sienra-Monge J-J, Wu H, Chiu GY, et al. Genome-wide association study implicates chromosome 9q21.31 as a susceptibility locus for asthma in mexican children. PLoS Genet. 2009;5:e1000623.

2. Lawrance-Owen AJ, Bargary G, Bosten JM, Goodbourn PT, Hogg RE, Mollon JD. Genetic association suggests that SMOC1 mediates between prenatal sex hormones and digit ratio. Hum. Genet. 2013;132:415–21.

3. GWAS Catalog [Internet]. [cited 2017 Nov 27]. Available from: http://www.ebi.ac.uk/gwas/api/search/downloads/ancestry

4. The World Factbook — Central Intelligence Agency [Internet]. [cited 2017 Aug 4]. Available from: https://www.cia.gov/library/publications/resources/the-world-factbook/index.html

5. GWAS Catalog [Internet]. [cited 2017 Nov 27]. Available from: http://www.ebi.ac.uk/gwas/search?query=GCST004794

6. Melén E, Granell R, Kogevinas M, Strachan D, Gonzalez JR, Wjst M, et al. Genome-wide association study of body mass index in 23 000 individuals with and without asthma. Clin. Exp. Allergy J. Br. Soc. Allergy Clin. Immunol. 2013;43:463–74.

7. Stacey SN, Sulem P, Jonasdottir A, Masson G, Gudmundsson J, Gudbjartsson DF, et al. A germline variant in the TP53 polyadenylation signal confers cancer susceptibility. Nat. Genet. 2011;43:1098–103.

8. Porras-Hurtado L, Ruiz Y, Santos C, Phillips C, Carracedo A, Lareu MV. An overview of STRUCTURE: applications, parameter settings, and supporting software. Front. Genet. 2013;4:98.

9. Alexander DH, Novembre J, Lange K. Fast model-based estimation of ancestry in unrelated individuals. Genome Res. 2009;19:1655–64.

10. Price AL, Patterson NJ, Plenge RM, Weinblatt ME, Shadick NA, Reich D. Principal components analysis corrects for stratification in genome-wide association studies. Nat. Genet. 2006;38:904–9.

11. Purcell S, Neale B, Todd-Brown K, Thomas L, Ferreira MAR, Bender D, et al. PLINK: a tool set for whole-genome association and population-based linkage analyses. Am. J. Hum. Genet. 2007;81:559–75.

12. Kang HM, Sul JH, Service SK, Zaitlen NA, Kong S-Y, Freimer NB, et al. Variance component model to account for sample structure in genome-wide association studies. Nat. Genet. 2010;42:348–54.

13. Wang S, Ray N, Rojas W, Parra MV, Bedoya G, Gallo C, et al. Geographic patterns of genome admixture in Latin American Mestizos. PLoS Genet. 2008;4:e1000037.

14. Galanter JM, Fernandez-Lopez JC, Gignoux CR, Barnholtz-Sloan J, Fernandez-Rozadilla C, Via M, et al. Development of a panel of genome-wide ancestry informative markers to study admixture throughout the Americas. PLoS Genet. 2012;8:e1002554.

15. Moreno-Estrada A, Gravel S, Zakharia F, McCauley JL, Byrnes JK, Gignoux CR, et al. Reconstructing the population genetic history of the Caribbean. PLoS Genet. 2013;9:e1003925.

16. Homburger JR, Moreno-Estrada A, Gignoux CR, Nelson D, Sanchez E, Ortiz-Tello P, et al. Genomic Insights into the Ancestry and Demographic History of South America. PLoS Genet. 2015;11:e1005602.

17. Cole AM, Cox S, Jeong C, Petousi N, Aryal DR, Droma Y, et al. Genetic structure in the Sherpa and neighboring Nepalese populations. BMC Genomics. 2017;18:102.

[13][14][15][16][17]
